# Are Emotional Eating and Other Eating Behaviors the Missing Link in the Relationship between Inadequate Sleep and Obesity? A Systematic Review

**DOI:** 10.3390/nu15102286

**Published:** 2023-05-12

**Authors:** María Fernanda Zerón-Rugerio, Sara Doblas-Faxeda, María Diez-Hernández, Maria Izquierdo-Pulido

**Affiliations:** 1 Nutrition and Food Safety Research Institute (INSA-UB), Torribera Campus, University of Barcelona, 08907 Barcelona, Spain; maria_izquierdo@ub.edu; 2 Department of Fundamental and Medical-Surgical Nursing, Faculty of Medicine and Health Sciences, Bellvitge Campus, University of Barcelona, 08907 Barcelona, Spain; 3 Department of Nutrition, Food Science, and Gastronomy, Food Science Torribera Campus, University of Barcelona, 08921 Barcelona, Spain; sdoblas.faxeda@gmail.com (S.D.-F.); mariadiezdh@gmail.com (M.D.-H.)

**Keywords:** sleep, obesity, emotional eating, eating behaviors, disinhibition, cognitive restraint, gender, stress

## Abstract

Evidence is emerging to explain that the relationship between inadequate sleep and obesity could be influenced by emotional eating and other eating behaviors such as disinhibition. Therefore, our aim was to conduct a systematic review to analyze the potential role of emotional eating and other eating behaviors in the relationship between inadequate sleep and obesity. We conducted a comprehensive search on 2 databases (Medline and Scopus) looking for records from 1 January 2011 until 31 December 2022 without language restrictions. Cross-sectional, longitudinal, and interventional studies were included if they assessed the association between sleep and emotional eating, as well as the role of emotional eating on the relationship between inadequate sleep and obesity. Secondary outcomes included studies analyzing the link between sleep and other eating behaviors as well as their role in the sleep–obesity relationship. Our results showed that emotional eating and disinhibition play a significant role in the relationship between inadequate sleep and obesity, especially in women. Furthermore, we provide evidence of other eating behaviors (e.g., external eating, eating competence, and hunger), which are also associated with poor sleep outcomes. However, these behaviors do not seem to be determinants of the association between sleep and obesity. In conclusion, our results suggest that individuals with inadequate sleep who are prone to emotional eating and/or disinhibition may require tailored approaches for obesity prevention and treatment.

## 1. Introduction

Inadequate sleep (characterized by short sleep duration [<6 h/day] and/or poor sleep quality) is highly prevalent in modern society [1,2]. Unfortunately, evidence has shown that inadequate sleep can act as a stressor for metabolic health and can lead to obesity [1]. Furthermore, several studies have shown that the pathway through which inadequate sleep can lead to obesity is related to “what” and “how much” people eat when they have not slept well [2,3,4,5]. The latter includes having more opportunities to eat due to increased wakefulness, alterations in appetite-regulating hormones (mainly ghrelin and leptin), and an increased sensitivity to food reward [2,3,4,5].

Noteworthy, Zuraikat et al. [2] recently reviewed this topic and provided evidence sustaining that higher appraisal of food reward is the most appropriate mechanism to explain the shift towards a poor diet quality after periods of inadequate sleep. As such, inadequate sleep has been linked to increased neuronal activity in response to the intake of highly palatable foods [6]. Not surprisingly, the results of a meta-analysis showed that after a night of short sleep duration, energy intake can increase up to ~350 kcal the next day [7]. Furthermore, inadequate sleep has been linked to adverse dietary behaviors, including lower adherence to healthy dietary patterns [2,5,8,9], as well as a decreased intake of healthy foods, including vegetables, fruits, and whole grains [2].

However, evidence is also emerging to explain that the relationship between inadequate sleep and obesity could be influenced by other factors, including emotional eating [9,10,11,12,13]. Konttinen [10] explained that inadequate sleep interferes with emotion regulation and may also affect the ability to effectively manage and respond to an emotional experience. In consequence, when sleep is neglected and people are unable to cope with emotionally negative situations (sadness, anger, anxiety, nervousness, etc.) they turn to food for comfort [9,14]. Note that eating sweet and fatty foods is known to alleviate behavioral signs of distress [15,16]. As such, emotional eating has been significantly associated with lower quality of the diet, as well as a higher consumption of fast food, commercially baked goods or pastries, sweets, and candy [9]. Furthermore, emotional eating has been highlighted as a significant mediator of the relationship between inadequate sleep and obesity [9,17,18].

Moreover, other studies have shown that other obesogenic eating behaviors related to the need to eat in the presence of disinhibited stimuli, such as emotional stress and/or palatable foods, could also play a role as mediators of the relationship between sleep and obesity [19,20]. In this line, Blumfield et al. [19] explained that, in the context of inadequate sleep, there is a greater drive for exciting rewards, which in combination with a high food-related disinhibition trait can exacerbate the food seeking behavior, especially for palatable foods. In consequence, disinhibition has also been associated with obesity and less healthy food choices [20,21].

Considering the aforementioned, in this systematic review, we aimed to analyze the potential role of emotional eating in the relationship between inadequate sleep and obesity. Furthermore, we studied other eating behaviors (e.g., disinhibition) that could also be determinants of the sleep–obesity association. To do so, we first provided an overview of the relationship between sleep and eating behaviors (including emotional eating). Subsequently, we studied whether emotional eating and other eating behaviors might be the missing link in the sleep–obesity relationship.

## 2. Materials and Methods

We conducted a comprehensive search on 2 databases (Medline and Scopus) looking for records from 1 January 2011 until 31 December 2022 without language restrictions. Additionally, we searched the reference lists for relevant literature. Search terms were determined in collaboration with all the authors through the exploration of key words in the literature. Search terms included the terms “sleep”, “emotional eating”, and “obesity” or “weight loss”. Then, a study protocol was prepared in accordance with the PRISMA guidelines, although it was not registered.

### 2.1. Study Inclusion and Exclusion Criteria

This systematic review included all studies that evaluated men and/or women without diagnosed chronic diseases, eating disorders, sleep conditions, or emotional disorders (such as depression or anxiety). We also included studies including people of all body mass index (BMI) ranges, aged ≥ 18–65 years. In addition, cross-sectional, longitudinal, and interventional studies were included if they assessed the association between sleep and emotional eating, as well as the role of emotional eating on the relationship between inadequate sleep and obesity. Secondary outcomes included studies analyzing the link between sleep and other eating behaviors (Table 1), as well as their role in the sleep–obesity relationship. The methodology that was used to assess inadequate sleep and eating behaviors was not pre-specified, since the literature provides no consensus regarding the most suitable tools/methodologies to assess these variables. Regarding the exclusion criteria, we did not include studies with patients who were undergoing chronic pharmacological therapies (i.e., statins for cholesterol treatments or antidepressants). In addition, we excluded reviews, meta-analyses, and editorials.

### 2.2. Data Extraction and Quality Assessment

First, two reviewers independently screened the titles and abstracts of identified studies for formal review and analysis. Subsequently, we retrieved full texts of the identified studies and extracted the data. When necessary, clarification from an independent reviewer was sought. The data extraction form was designed based on Cochrane guidelines and included author name and year, country, study design, number of participants, gender, age, BMI, methods for sleep and eating behavior assessment, and key findings (considering the significance in the correlation coefficients, β coefficients, and *p*-values). All the data obtained in the study were independently extracted.

Subsequently, we assessed the quality of the included studies using the Quality Criteria Checklist for primary research proposed by the Academy of Nutrition and Dietetics [25]. Briefly, this tool assesses the quality of a study based on inclusion/exclusion criteria, risk of bias, and generalizability as well as data collection and analysis. According to the 10-item Quality Criteria Checklist, article quality can be rated as “negative” (if 6 responses to validity questions were “no”), “neutral” (if the responses to validity questions 2, 3, 6, and 7 were “no”), and “positive” (if the majority of responses to validity questions 2, 3, 6, and 7 were “yes” and at least an additional “yes”).

## 3. Results

**Figure 1:**
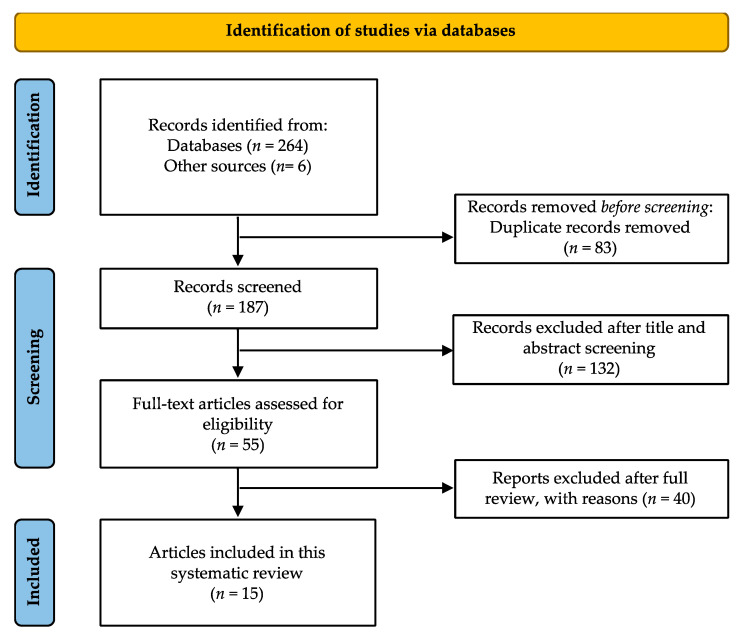
Flow chart of literature searching, screening, and selection of articles for inclusion in this systematic review.

A total of 270 articles were retrieved from the initial electronic search (Figure 1). After removing duplicates, we identified 187 publications for screening, of which 55 were retrieved in full text for further evaluation. Based on the selection criteria, a total of 15 articles were suitable for inclusion in this systematic review. It should be noted that the results presented in this research work include a sample size of 11,698 participants, of whom 61.2% were women. In terms of the study designs, the majority (81%) of the studies included were cross-sectional [9,12,13,14,19,26,27,28,29,30], while the remaining 19% of the studies were longitudinal [17,18,20]. The key findings of all studies included in this systematic review are shown in Table 2.

### 3.1. Associations between Emotional Eating and Other Eating Behaviors and Sleep

Overall, the results of the studies included in this systematic review showed that poor sleep quality was significantly associated with emotional eating [9,12,14,30]. In addition, we observed that other eating behaviors such as external eating [14], low eating competence [24,31], greater hunger [19], disinhibited eating behaviors [9,19,30], and higher cognitive restraint [9,26] were significantly associated with poor sleep quality.

Regarding sleep duration, findings from three longitudinal studies showed that short sleep duration was significantly associated with greater emotional eating [10,18] and disinhibited eating behavior [20]. As for other sleep quality parameters, Ghani et al. [27] observed that increased insomnia symptoms were significantly associated with higher emotional eating and greater uncontrolled eating behaviors.

### 3.2. The Role of Emotional Eating and Other Eating Behaviors as Mediators of the Relationship between Sleep and BMI

According our findings, the results derived from three studies (one cross-sectional and two longitudinal studies) that emotional eating mediated the relationship between inadequate sleep and obesity [9,17,18], especially in women [17,18]. Regarding the role of other eating behaviors in the sleep–obesity relationship, the results of two studies highlighted that disinhibited eating behavior was a significant mediator of the relationship between inadequate sleep and obesity [19,20]. Noteworthy, Chaput et al. [20] observed that the risk of developing overweight/obese status was higher among short duration sleepers with high disinhibited eating behavior (OR: 4.49 [95% CI: 3.06–6.06]).

Among other eating behaviors that can play a role in the relationship between sleep and obesity, we found two cross-sectional studies that evidenced the role of cognitive restraint as a mediator/moderator of the association between poor sleep quality and BMI [9,26]. On the one hand, Barragán et al. [26] observed that cognitive restraint attenuated the relationship between sleep fragmentation (a marker of poor sleep quality) and BMI by making the slope less negative. On the other hand, our research group recently showed that, in young adults, cognitive restraint mediated the relationship between poor sleep quality and obesity [9]. However, it should be noted that these conclusions were drawn out of two studies whose study populations and methodologies to assess sleep quality were different [9,26].

### 3.3. Other Determinants in the Association between Sleep, Eating Behaviors, and Obesity

Among other significant findings, the results derived from this systematic review showed that stress can be a potential confounder in the relationship between inadequate sleep and eating behaviors [12,14]. In this regard, Dweck et al. [14] showed that food consumption under stress condition was higher in the normal sleep group but did not differ in the short sleep group. Moreover, another cross-sectional study pointed out that at higher stress levels, individuals with good sleep quality reported higher scores for uncontrolled eating than individuals with poor sleep quality [28].

Furthermore, we observed that another factor that can play a significant role in the relationship between sleep and emotional eating is gender. As such, one study showed significant interactions between being a women and having greater emotional eating behavior [12]. Consistently, the results of two longitudinal studies showed that gender was a determinant in the sleep–obesity relationship [17,18]. The studies revealed that women, but not men, showed a two-way emotional interaction between emotional eating and sleep duration on BMI change (*p* < 0.05) [17,18]. Furthermore, Barragán et al. [26] showed that men with poor sleep quality showed greater hunger levels.

### 3.4. Quality Assessment

The results derived from the quality assessments are shown in Table 3. Overall, the quality of 73% of the studies included was positive, while the quality of the remaining 17% of the articles was rated as neutral, due to selection bias and the lack of appropriate adjustments to account for confounding factors.

## 4. Discussion

The main finding of this systematic review is that emotional eating plays a significant role in the relationship between inadequate sleep and obesity [9,17,18]. As such, our results show that the interplay between poor sleep outcomes and emotional eating are associated with the subsequent development of obesity, especially in women [17,18]. In addition, based on the collected evidence, disinhibition is another eating behavior that could also be a significant mediator of the sleep–obesity relationship [19,20]. Furthermore, we provide evidence of other eating behaviors, such as external eating [14], eating competence [24,31], and hunger [19], which are also associated with poor sleep outcomes. However, these behaviors [14,19,24,31] do not seem to be determinants of the association between poor sleep quality and obesity. Among other findings, the studies reviewed in this research work revealed that stress and gender, are important factors to consider when studying the associations between sleep, eating behaviors, and obesity.

To our knowledge, this is the first systematic review to provide evidence regarding the role of emotional eating on the relationship between inadequate sleep and obesity. As such, evidence shows that when individuals have not slept well (in quantity or quality), they are more likely to face negative situations by turning to food for comfort as a coping mechanism [9,12,14,17,18,30]. However, emotional eating is a maladaptive behavior, since it is unlikely to result in long-term improvements in mood [17]. Instead, emotional eating is sometimes followed by negative emotions, such as feelings of guilt, that could lead to overeating episodes [17,32]. Furthermore, Martin-García et al. [22] pointed out that overeating episodes (e.g., emotional eating) could lead to weight gain, and that this weight increase could lead to caloric restriction, which also alternates with episodes of overeating, becoming a vicious cycle [22,33]. As such, some studies have shown that restrictive eating behaviors could be associated with higher BMI [34,35].

In this sense, one of the cross-sectional studies included in this review showed that both emotional eating and cognitive restraint were significant mediators of the relationship between poor sleep quality and obesity among young adults [9]. In this case, the authors postulated that poor sleep quality could increase the risk of overeating in response to emotional stress, leading young adults to make a conscious effort to control food intake as a strategy to manage their body weight [9]. However, it is plausible that age can influence the role of cognitive restraint in the sleep–obesity relationship. Note that another study, conducted in a smaller and more heterogeneous sample, showed that the association between poor sleep quality and BMI was moderated by cognitive restraint, making the slope less negative [26]. According to the authors, this could imply that individuals with a high dietary restraint may be at lower risk for weight gain in response to poor sleep [26]. It should be noted that this inconclusiveness could be due to the heterogeneity in the study populations (young adults aged 18–30 years [9] vs. adults aged 20–73 years [26]) as well as in the methodologies that were used to assess the quality of sleep (PSQI [9] vs. actigraphy [26]). Therefore, further studies are needed to elucidate the role of cognitive restraint in the relationship between sleep and obesity and whether age can be an important factor to consider.

This systematic review also revealed that disinhibition is another eating behavior that could be relevant in the sleep–obesity relationship [19,20,30]. Interestingly, the results of a 6-year longitudinal study showed that short sleepers (<6 h/day) that had a high disinhibition eating trait were more likely to overeat and gain weight [20]. Furthermore, this study showed that energy intake was significantly higher in short sleepers with a high disinhibition score [20]. This could be due to the fact that individuals with highly disinhibited eating behavior have a higher liking for sweet and high-fat foods [2,9,14,19,20,36]. In addition, those with a high disinhibited eating behavior showed a higher wanting of food, stronger food cravings, and a lower willingness to control eating behavior [21].

In line with the aforementioned, three cross-sectional studies observed significant associations between inadequate sleep and uncontrolled [9,27] and external eating [14]. Of which, one study revealed that uncontrolled eating was significantly associated with lower adherence to the Mediterranean diet as well as an increased attendance to fast-food restaurants and daily consumption of sweets [9]. These findings also support the findings of Zuraikat et al. [2] who highlighted the hedonic value of food intake as one of the most suitable mechanisms to explain the shift towards a poor diet quality after periods of inadequate sleep.

Moreover, findings from the studies of Quick et al. [24,31] suggested that participants who were low competent eaters were more likely to have poor sleep quality and higher BMI. Note that eating competence is a behavior that emphasizes eating enjoyment and internal regulation of food intake. Accordingly, individuals with lower eating competence are less likely to trust internal regulation processes [24,31]. Consequently, evidence has shown that having lower eating competence has been associated with lower diet quality [37].

Among other findings, our results also shed light on the role that stress could play in the relationship between inadequate sleep and eating behaviors [12,14]. Furthermore, some authors have suggested that short sleep duration can act as a type of stressor that is especially likely to increase food intake in emotional eaters [14,17]. In this line, results from the only experimental study included in this systematic review showed that food consumption under a stress condition was higher in the normal sleep group but did not differ in the short sleep group [14]. The authors hypothesized that short sleep might produce an effect on eating behavior that is similar to the stress condition. Meanwhile, according to the authors, the results obtained in short sleepers suggested that there was a ceiling in the amount of food consumed when stressed, therefore there was no additive effect for short sleepers [14].

Moreover, another cross-sectional study showed that, at higher stress levels, individuals with good sleep quality reported higher scores for uncontrolled eating compared with participants with poor sleep quality [28]. Interestingly, Yau and Potenza [38] explained that stress is associated with higher glucocorticoids levels, which can intensify emotions and motivation. As such, and given the rewarding properties of food, it is plausible that hyperpalatable foods may serve as a “comfort food” that can act as a form of self-medication to dispel unwanted stress [38].

Likewise, Richards et al. [12] observed that that high stress levels and the lack of sleep affected cognitive restraint in freshmen (18 years) more than older students. In this case, freshmen students had more cognitive restraint when perceived stress levels were high and less cognitive restraint when perceived stress levels were low. Note that dietary restraint has been shown to exacerbate eating in response to food cues and stress [38]. This may explain why highly restrained eaters increase food intake during stressful conditions [38].

Furthermore, we found that gender is another factor to consider when studying the relationship between sleep, eating behaviors, and obesity [12,17,31]. As such, results drawn from two longitudinal studies found that emotional eating was a significant mediator of the relationship between sleep and obesity in women, but not in men [17,18]. Konttinen et al. [17] explained that women were more prone to emotional eating than men due to the physiological response to stress. Accordingly, women often show lower hypothalamic–pituitary–adrenal (HPA) axis and autonomic stress responses than men of the same age [17,39], except in the luteal phase of the menstrual cycle when cortisol levels approach those of men [39]. It is important to consider that the physiological response to stress is the hyper-activation of the HPA axis and the decrease in appetite [38,39], which could partly explain why women are more likely to cope with emotional stress with food.

Regarding the interaction between gender and other eating behaviors, one study showed that men with poor sleep quality showed greater hunger levels [26]. This result aligns with the findings of St-Onge et al. [40] who demonstrated that, under sleep-restriction conditions (4 h in bed), men had increased fasting and morning ghrelin concentrations while no such associations were found in women.

### Limitations

Although our systematic review provides a comprehensive overview of the role of emotional eating and other eating behaviors in the relationship between sleep and obesity, there are certain limitations with regard to interpreting our findings. The first is the observational and longitudinal nature of the studies included. Second, we recognize the heterogeneity in the methods that were used to evaluate eating behavior as a limitation. Note that these differences could limit the generalizability of our findings, especially considering that eating behavior characteristics may differ depending on which questionnaire was used. Another limitation is that the majority of the studies evaluated sleep quality and duration through questionnaires, rather than objective measures such as actigraphy. Finally, it is important to consider that the relationship between sleep and obesity is complex and bidirectional. As such, other chronic conditions (e.g., obstructive sleep apnea) [41], emotional states (e.g., depression or anxiety), and lifestyle habits [1], could also play a role in the sleep–obesity association.

## 5. Conclusions

The findings of this systematic review provide a novel perspective on the role of eating behaviors in the relationship between inadequate sleep and obesity. Accordingly, a growing body of evidence has shown that emotional eating and disinhibition are two eating behaviors that are significantly associated with poor sleep outcomes (quality and duration) as well as with higher BMI. However, these conclusions were drawn from cross-sectional and longitudinal studies. Therefore, our results open a new framework for future intervention studies, as they suggest that individuals with inadequate sleep who are prone to emotional eating and/or disinhibition may require personalized approaches for obesity prevention and treatment. In addition, our findings shed light on cognitive restraint as an eating behavior that could be a determinant of this relationship. Finally, this research work showed that other factors, such as stress, gender, and age, could be determinant in the relationship between sleep, eating behaviors, and obesity.

## Figures and Tables

**Table 1:** Definitions of the eating behaviors that were included in this systematic review.

Eating Behavior	Definition
Cognitive restraint	The individual’s conscious efforts to control what they eat in order to keep or lose weight [21,22].
Disinhibition	The need to overeat in the presence of palatable foods or other disinhibiting stimuli such as emotional stress [19].
Eating competence	Emphasizes eating enjoyment, internal regulation of food intake and letting body weight be dictated by lifestyle and genetics, using skills to provide meals regularly, and eating a variety of foods for pleasure, rather than to meet dietary guidelines alone [23,24].
Emotional eating	The need to overeat when individuals are unable to cope with emotionally negative situations [22].
External eating	Eating in response to the presentation of food regardless of hunger [14].
Hunger	The extent to which hunger feelings are perceived and the extent to which such feelings then evoke food intake.
Uncontrolled eating	Expresses the tendency to eat excessively in response to the loss of control over food itself [22].

**Table 2:** Summary of cross-sectional studies included in this systematic review.

Author (Region, Year)	Population Studied (*n*, Age, Gender, BMI)	Study Design	Intervention	Sleep Assessment Methods	Eating Behavior Assessment Methods	Key Findings
Chaput JP, et al. (Canada, 2011) [20]	*n* = 276 21–64 years 53.3% women 52.4% overweight/obese	Longitudinal study (6 years)	No intervention	On average, how many hours do you sleep a day?	TFEQ	The risk of becoming overweight/obese was higher for short duration sleepers with high disinhibited eating behavior (OR: 4.49 [95% CI: 3.06–6.06]).
Dweck JS, et al. (USA, 2014) [14]	*n* = 184 18.6 ± 0.1 years 100% women 22.7 ± 0.3 kg/m^2^	Cross-sectional	No intervention	Sleep quality index	DEBQ	Poorer sleep quality was associated with elevated emotional eating (*p* = 0.004) and external eating (*p* = 0.005) scores but not with dietary restraint. However, BMI was not correlated with sleep quality.
	*n* = 64 18.8 ± 0.4 years 100% women 24.5 ± 0.6 kg/m^2^	Experimental	Participants were asked to solve an unsolvable Sudoku puzzle to induce stress.	PSQI	DEBQ	Short sleep (<7h/day) predicted higher food consumption in high emotional eaters in the control condition. Food consumption under stress condition was higher in the normal sleep group but did not differ in the short sleep group.
Van Strien T, et al. (Netherlands, 2014) [18]	*n* = 1464 44.2 ± 8.9 years 37.8% women 25.3 ± 3.7 kg/m^2^	Longitudinal study (1 year)	No intervention	On average, how many hours do you sleep a day?	DEBQ	Women showed a two-way emotional interaction between emotional eating and sleep duration on BMI change (*p* < 0.05). No such associations were found in men.
Quick V, et al. (USA, 2014) [31]	*n* = 1252 18–24 years 59% women 23.6 ± 3.7 kg/m^2^	Cross-sectional	No intervention	PSQI	TFEQ Eating Competence Satter Inventory	Short sleep duration (*p* < 0.001), greater sleep disturbances (*p* = 0.047), higher sleep latency (*p* = 0.008), and higher PSQI score (*p* < 0.001) were more common in overweight/obese participants. Overweight/obese participants were also more likely to have greater cognitive restraint (*p* < 0.001), emotional eating (*p* = 0.032), and uncontrolled eating (*p* = 0.005) than participants with a healthy weight. PSQI scores and low eating competence were significant predictors of overweight/obese status.
Quick V, et al. (USA, 2015) [24]	*n* = 1035 18–24 years 61% women 23.5 ± 3.6 kg/m^2^	Cross-sectional	No intervention	PSQI	Eating Competence Satter Inventory	A higher proportion of those in the eating competence (EC) group reported adequate sleep quality (67% vs. 57% in non-EC, *p* = 0.001), sleep duration of ≥7 h nightly (58% vs. 50% in non-EC, *p* = 0.007), and infrequent daytime dysfunction (72% vs. 65% in non-EC, *p* = 0.02). PSQI, eating competence score, age and gender (female) were significantly (*p* < 0.01) associated with overweight/obese status.
Blumfield ML, et al. (USA, 2018) [19]	*n* = 602 38.9 ± 14.5 years 64.3% women 27.8 ± 6.0 kg/m^2^	Cross-sectional	No intervention	PSQI	TFEQ	Poor sleep quality was associated with greater hunger (*p* = 0.030) and higher disinhibited eating (*p* < 0.001). In addition, higher disinhibited eating behavior was associated with higher BMI (*p* < 0.001). Disinhibition mediated the relationship between sleep quality and BMI (*p* = 0.001).
Arslan M, et al. (Turkey, 2019) [13]	*n* = 535 18–65 years 63% women 58.5% overweight/obese	Cross-sectional	No intervention	PSQI	Emotional appetite questionnaire Eating attitudes test	There was a significant relationship between BMI and poor sleep quality (*p* = 0.040). The group with the highest rate of disrupted eating attitudes had high BMI levels (*p* < 0.001). A high BMI was associated with higher negative emotional appetite scores (*p* < 0.001).
Konttinen H, et al. (Finland, 2019) [17]	*n* = 3735 53.0 ± 13.0 years 54.8% 26.8 ± 4.7 kg/m^2^	Longitudinal (7 years)	No intervention	How many hours do you usually sleep at night?	TFEQ-R18	Emotional eating behavior predicted a higher 7-year increase in BMI (*p* = 0.007) and waist circumference (*p* = 0.026) in women. Short sleep duration moderated the relationship between emotional eating and BMI. As such, emotional eating predicted BMI (*p* = 0.007) and waist circumference gain (*p* = 0.026) in short sleepers.
Richards AL, et al. (USA, 2020) [28]	*n* = 162 43.6 ± 1.0 years 92.0% women 33.6 ± 0.5 kg/m^2^	Cross-sectional	No intervention	How many hours of sleep do you get on average per night? Over the last month, how would you rate your sleep quality?	TFEQ-R18	The association between hours of sleep and emotional eating did not reach statistical significance (*p* = 0.070). Sleep quality was found to modify the relationship between uncontrolled eating and stress (*p* = 0.040). Consequently, at higher stress levels, individuals with good sleep quality reported higher scores for uncontrolled eating than individuals with poor sleep.
Papaconstantinou E, et al. (Canada, 2020) [29]	*n* = 245 43.6 ± 1.0 years 86.1% women 33.6 ± 0.5 kg/m^2^	Cross-sectional	No intervention	PSQI Sleep duration	TFEQ-R18	A non-significant trend was found between non-compliance with sleep recommendations (7–9 h/day) and emotional eating behavior (*p* = 0.062).
Eck KM, et al. (USA, 2020) [30]	*n* = 535 32.3 ± 5.8 years 100% women 29.0 ± 8.6	Cross-sectional	No intervention	PSQI	TFEQ Adventurous Eating Scale	Women with poor sleep quality had higher BMI (*p* = 0.001) and more emotional (*p* = 0.001) and disinhibited eating (*p* = 0.028), compared to women with moderate or high sleep quality.
Richards AL, et al. (USA, 2021) [12]	*n* = 153 20.7 ± 4.8 years 71.6% women 26.1 ± 5.9 kg/m^2^	Cross-sectional	No intervention	PSQI	TFEQ-R18	Emotional eating, but not uncontrolled eating or cognitive restraint, was significantly associated with PSQI (*p* = 0.020). Freshmen reporting fewer hours of sleep had lower cognitive restraint scores than men with more hours of sleep (*p* = 0.040)
Barragán R, et al. (USA, 2021) [26]	*n* = 179 36.0 ± 13.1 years 68.7% women 26.6 ± 3.4 kg/m^2^	Cross-sectional	No intervention	Actigraphy	TFEQ	Higher wakefulness after sleep onset, higher sleep fragmentation index, and lower sleep efficiency were associated with higher dietary restraint (*p* = 0.007, *p* = 0.041, *p* = 0.010, respectively). In addition, higher dietary restraint attenuated the positive relationships between sleep fragmentation index and BMI (*p* = 0.034). Men with lower sleep efficiency, greater sleep latency, and higher sleep fragmentation showed a greater tendency towards hunger (*p* = 0.009, *p* = 0.020, *p* = 0.003).
Zerón-Rugerio MF, et al. (Spain, 2022) [9]	*n* = 925 21.4 ± 2.5 years 77.8% women 21.8 ± 3.1 kg/m^2^	Cross-sectional	No intervention	PSQI	TFEQ-R21	Emotional eating (*p* < 0.0001), cognitive restraint (*p* < 0.001), and uncontrolled eating (*p* < 0.0001) were significantly associated with sleep quality. BMI was significantly associated with PSQI score (*p* = 0.021), emotional eating (*p* < 0.0001), and cognitive restriction (*p* < 0.0001). Emotional eating and cognitive restraint were significant mediators of the association between sleep quality and BMI.
Ghani SB, et al. (USA, 2022) [27]	*n* = 100 36.5 ± 19.1 years 47% women 30.2 ± 6.1 kg/m^2^	Cross-sectional	No intervention	Insomnia Severity Index PSQI Epworth Sleepiness Scale Sleep timing questionnaire	TFEQ-R18	Uncontrolled eating and emotional eating were associated with poorer sleep quality (*p* < 0.05), daytime sleepiness (*p* < 0.05), shorter weekend sleep duration (*p* < 0.05), and greater insomnia severity (*p* < 0.05).

BMI: body mass index; DEBQ: Dutch eating behavior questionnaire; EC: eating competence; PSQI: Pittsburg sleep quality index; TFEQ: Three-Factor Eating Questionnaire; TFEQ-R18: Three Factor Eating Questionnaire—Revised version (18 items); TFEQ-R21: Three Factor Eating Questionnaire—Revised version (21 items); USA: United States of America.

**Table 3:** Quality assessment of quantitative articles.

Author (Year)	1	2	3	4	5	6	7	8	9	10	Total	Quality
Chaput JP, et al. (2011) [20]	+	+	+	+	-	+	+	+	+	+	9/10	Positive
Dweck JS, et al. (2014) [14] ^a^	+	+	-	-	-	+	+	+	+	+	7/10	Neutral
Dweck JS, et al. (2014) [14] ^b^	+	+	-	+	-	+	+	+	+	+	8/10	Neutral
Van Strien T, et al. (2014) [18]	+	+	+	+	-	+	+	+	+	+	9/10	Positive
Quick V, et al. (2014) [31]	+	+	+	+	-	+	+	+	+	+	9/10	Positive
Quick V, et al. (2015) [24]	+	+	+	+	-	+	+	+	+	+	9/10	Positive
Blumfield ML, et al. (2018) [19]	+	+	+	+	-	+	+	+	+	+	9/10	Positive
Arslan M, et al. (2019) [13]	+	+	-	-	-	+	+	+	+	+	7/10	Positive
Konttinen H, et al. (2019) [17]	+	+	+	+	-	+	+	+	+	+	9/10	Positive
Richards AL, et al. (2020) [28]	-	+	+	+	-	+	+	+	+	+	8/10	Positive
Papaconstantinou E, et al. (2020) [29]	+	-	+	-	-	+	+	+	+	+	7/10	Neutral
Eck KM, et al. (2020) [30]	+	+	-	+	-	+	+	+	+	+	8/10	Neutral
Richards AL, et al. (2021) [12]	+	+	+	+	-	+	+	+	+	+	9/10	Positive
Barragán R, et al. (2021) [26]	+	+	+	+	-	+	+	+	+	+	9/10	Positive
Zerón-Rugerio MF, et al. (2022) [9]	+	+	+	+	-	+	+	+	+	+	8/10	Positive
Ghani SB, et al. (2022) [27]	+	-	+	-	-	+	+	+	+	+	7/11	Neutral

^a^ Quality assessment of experiment 1 (cross-sectional study). ^b^ Quality assessment of experiment 1 (experimental protocol). +, The criterion was clearly satisfied; -, The criterion was not clearly satisfied. Validity questions: 1 Was the research question clearly stated? 2 Was the selection of study subjects/patients free from bias? 3 Were study groups comparable? 4 Was method of handling withdrawals described? 5 Was blinding used to prevent selection bias? 6 Were intervening factors described? 7 Were outcomes clearly defined and the measurements valid and reliable? 8 Was the statistical analysis appropriate for the study design and type of outcome indicators? 9 Are conclusions supported by results with biases and limitations taken into considerations? 10 Is bias due to study’s funding or sponsorship unlikely? Quality was rated as negative (if 6 responses to validity questions were “no”), neutral (if the responses to validity questions 2, 3, 6, and 7 were “no”), and positive (if the majority of responses to validity questions 2, 3, 6, and 7 were “yes” and at least an additional “yes”).
